# Care of neonates following in-utero growth restriction: A prospective cohort study exploring neonatal morbidity

**DOI:** 10.1038/s41372-025-02397-9

**Published:** 2025-08-21

**Authors:** M. G. Alda, A. G. Wood, T. MacDonald, J. K. Charlton

**Affiliations:** 1 Department of Paediatrics, University of Melbourne, Melbourne, VIC Australia; 2 Department of Paediatrics, Mercy Hospital for Women, Melbourne, VIC Australia; 3 School of Psychology, Deakin University, Melbourne, VIC Australia; 4 Clinical Sciences, Murdoch Children’s Research Institute, Melbourne, VIC Australia; 5 Department of Neonatal Medicine, Royal Children’s Hospital, Melbourne, VIC Australia; 6 Department of Obstetrics, Gynaecology and Newborn Health, University of Melbourne, Melbourne, VIC Australia; 7 Mercy Perinatal, Mercy Hospital for Women, Melbourne, VIC Australia; 8 Division of Neonatology, BC Women’s Hospital, Vancouver, BC Canada; 9 Department of Pediatrics, University of British Columbia, Vancouver, BC Canada

**Keywords:** Outcomes research, Disease model

## Abstract

**Objective:**

This study compared neonatal morbidities in Small-for-Gestational-Age (SGA) preterm infants to gestation- and birthweight-matched controls.

**Methods:**

Prospective cohort study conducted on preterm infants born between 24^+0^ and 36^+6^ weeks of gestation at an Australian NICU. SGA infants (birthweight <10th centile) were matched with same-sex, well-grown controls (birthweight ≥10th centile) based on gestation or birthweight. Neonatal clinical data, including growth and morbidities, were compared.

**Results:**

Among 148 preterm infants (54 SGA, 54 in each control group). SGA infants had higher rates of hypothermia, necrotising enterocolitis (NEC), and hypoglycaemia compared to controls. Intraventricular haemorrhage was more prevalent in birthweight-matched controls. SGA infants regained birthweight faster (day 8 vs. day 11, *p* < 0.0002). No significant differences were found in length of stay or respiratory support.

**Conclusion:**

SGA is an independent risk factor for hypothermia, NEC, and hypoglycaemia, beyond the risks incurred by being born preterm but well-grown for gestation.

## Background

Foetal growth restriction (FGR) defines a foetus who does not reach their biological growth potential [1]. It is a common pregnancy complication associated with increased risks of neonatal mortality and morbidity in the short and long term [2]. The term ‘small for gestational age (SGA)’ describes infants with a birthweight below the 10^th^ percentile for their gestational age. Although both terminologies (FGR and SGA) are commonly used interchangeably in the literature, not all FGR infants are born SGA and not all SGA infants are truly growth-restricted [3]. But while many term SGA infants are thought to be “constitutionally small”, the same description is generally not used in the preterm population.

Premature infants are more commonly growth-restricted because preterm birth is often due to perinatal pathologies that affect foetal growth [4–6]. Thus, when preterm infant birthweights are used to derive growth curves at preterm gestations, SGA infants are systematically underdiagnosed [7, 8]. Rather, SGA infants are over-represented amongst the premature population – but this is only appreciated if *in utero* growth charts are used, based on healthy fetuses with continuing pregnancies. Our previous retrospective study of tertiary neonatal intensive unit (NICU) inpatients [9] reflect the much lower tenth centile weight cutoffs on the Fenton charts [10] compared to in-utero charts used antenatally to diagnose FGR based on ultrasound estimated foetal weight.

Preterm birth frequently coexists with FGR, but timing of delivery in cases of FGR can be a serious dilemma for obstetricians. Preterm delivery will expose neonates to morbidities associated with immaturity, but these must be weighed against the risks of worsening foetal hypoxia and increasing stillbirth risk, should delivery be delayed. It is not surprising that while detection of FGR reduces the risk of stillbirth, increased neonatal morbidities are instead seen [11].

Here, we aimed to prospectively explore the trajectory of SGA preterm neonates admitted to a tertiary NICU. We aimed to evaluate comorbidities occurring due to SGA, accounting for the impact of prematurity. To do this, our SGA cohort was compared with two specific control groups of infants classified as appropriate-for-gestational-age (AGA): one matched for gestational age, and one matched for birthweight. We aimed to identify patterns of morbidity and care needs that were more strongly associated with either SGA, or prematurity. We hypothesised that some morbidities would be more prevalent among the SGA cohort, and that for others, gestation at birth would be the greatest risk factor, rather than birthweight centile.

## Methods

### Study population

Study participants were premature infants (24^+0^ and 36^+6^ weeks of gestation) born at Mercy Hospital for Women (MHW) in Melbourne, Australia, between April 2023 and February 2024. Infants were enroled as either SGA or AGA based on birthweight centile assigned by the Gestation Related Optimal Weight (GROW) customised growth chart [12] (www.gestation.net). GROW generates a ‘term optimal weight’ and then adjusts for factors which influence foetal growth to produce a centile according to the exact day of gestation – known to be important to best detect pathological growth associated with poor outcomes [13]. We adjusted for infant sex, and maternal height and weight. The SGA group included all enroled preterm infants with birthweight less than the 10^th^ percentile on the GROW chart. The control group consisted of AGA preterm infants (birthweight 10^th^ - 90^th^ percentile). The control group was divided into two groups: (i) infants matched to cases based on their gestational age (but with higher birthweights); and (ii) infants matched to cases by birthweight (which meant they were of lower gestational ages). Twins and triplets were eligible for inclusion as cases or controls, and their birthweight centile was calculated according to the same chart.

We excluded:

- Outborn infants transferred to MHW after birth and infants discharged home and readmitted to the nursery.
- Infants born with a birthweight above the 90^th^ percentile; as the largest babies also have increased risks of adverse perinatal outcomes, as seen in large epidemiological studies [14].
- Infants born at less than 24 weeks gestation; GROW does not calculate centiles at these gestations.
- Infants with diagnoses of any genetic condition known to impact either growth or neurodevelopment.

### Data collection

Maternal and infant demographic data were collected from medical records. We recorded if there was an antenatal diagnosis of FGR based on an ultrasound estimated foetal weight <10^th^ percentile. We collected data on *in utero* Doppler abnormalities of the umbilical arteries (UA), ductus venous (DV) and middle cerebral artery (MCA).

### Outcome measures

We used the Fenton Charts to assess neonatal growth during the hospital stay. We recorded weekly length, head circumference and weight, from birth to hospital discharge. We calculated the difference between the z-score at discharge and the z-score at birth. We applied the research bulk calculator from The University of Calgary, which uses completed weeks for weight, head circumference, and length to generate the z-scores [10]. We recorded baseline heart rate on day 1 of life and weekly until eight weeks of life or until discharged. If performed through clinical care, we also recorded blood gases to measure pH, lactate, and the lowest blood sugar level (BSL) on day one and the first week of life. Hypoglycaemia was defined as a BSL level less than 2.6 mmol/L.

The neonatal morbidities recorded were:

- Bronchopulmonary dysplasia (BPD), defined as oxygen requirement at 36 weeks of corrected post-menstrual age.
- Any grade of intraventricular haemorrhage (IVH) on ultrasound.
- Periventricular leukomalacia (PVL) on ultrasound.
- Any degree of retinopathy of prematurity (ROP).
- Proven NEC: (1) diagnosis at surgery or postmortem; (2) radiological diagnosis (pneumatosis intestinalis, portal vein gas, or a persistent dilated loop on serial X-rays) and a clinical history; or (3) clinical diagnosis, a clinical history plus abdominal wall cellulitis and palpable abdominal mass.

We measured the length of hospital stay (days) and the post-menstrual age at discharge (in weeks). Finally, the outcome of ‘death’ refers to death before discharge from the hospital.

### Statistical analysis

Descriptive data included mean ( ± standard deviation (SD)) and median (interquartile range (IQR)) for parametric and non-parametric data, respectively. Categorical data were compared between the study group (SGA) and the control (AGA) groups using the Chi-square test and Fisher’s exact test as appropriate. Continuous variables were compared between groups using t-tests or Wilcoxon Rank-Sum tests according to distribution of data.

We used logistic regression for the analysis of the NICU outcomes in each matched control group, we presented the data as odds ratios and 95% confidence interval. Results were considered statistically significant when the *p* = ≤ 0.05. Statistical analysis was performed using STATA (version 18).

## Results

A total of 222 infants met the inclusion criteria, including 72 whose parents declined to participate in the study. The remaining 150 infants (56 SGA and 54 in each of the control groups) were initially included. Two SGA infants had a postnatal diagnosis of Trisomy 21, and their data were excluded from the analysis. In summary, 54 SGA and 54 infants in each matched-control group were analysed in the study.

**Fig. 1:**
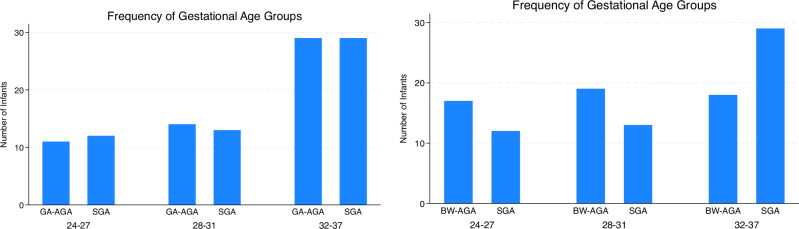
Distribution of gestational age within the groups. Y-axis displays the number of Infants that were, and X-axis displays three gestational age groups: extremely preterm (24 to 27 weeks), very preterm (28 to 31 weeks) and moderate to late preterm (32 to 37 weeks). *GA-AGA*, Gestational matched adequate for gestational age;*BW-AGA*, Birthweight matched adequate for gestational age; *SGA,* Small for gestational age.

Maternal characteristics (Table 1) were similar between the SGA and control groups. The SGA cohort had higher rates of ultrasound diagnosed FGR and pre-eclampsia. Among the SGA cohort, 37 had antenatal Doppler assessments, with 75.6% showing UA abnormalities, 46.4% with MCA abnormalities, and 10.3% with DV abnormalities. No Doppler abnormalities were noted in controls. Around one-third of the participants were multiple births, proportionally distributed within the groups. The infants (Table 1) in the SGA study group, had lower mean birthweights (1327 (547) grams) when compared to gestation-matched controls (1838 (606) grams). In contrast, they had higher gestational ages (31.3 (3.2) weeks) when compared to the birthweight-matched controls (29.6 (3.5) weeks). Gestational age distribution for each matched group was described in Fig. 1. Of all included in the SGA cohort, 43 (80%) had a birthweight less than the 3^rd^ centile on the GROW chart, which would categorise them as severely growth-restricted.

**Table 1:** Maternal and Infant demographics.

	SGA	GA matched CONTROLS		BW matched CONTROLS	
Number of infants	54	54	*p*	54	*p*
**Maternal Age, years, mean (SD)**	32.1 (4.8)	32.5 (5.2)	0.66	32.6 (4.9)	0.58
**Maternal BMI (kg/m**^**2**^**), mean (SD)**	26.7 (4.3)	25.1 (4.9)	0.07	26.0 (5.8)	0.45
**Maternal Ethnicity,** ***n*** **(%)**			0.24		0.37
Caucasian	31(57.4)	38 (70.3)		38 (70.3)	
Aboriginal	3 (5.5)	0		1 (1.8)	
Asian	15 (27.7)	12 (22.2)		13 (24.0)	
Other	5 (9.2)	4 (7.4)		2 (3.7)	
**US diagnosis of FGR,** ***n*** **(%)**	37 (68.5)	2 (3.7)	<0.001	3 (5.5)	<0.001
**Time of US diagnosis of FGR in weeks, mean (SD)**	27 (4.05)	33 (1.41)	0.07	34 (2)	0.01
**Antenatal Steroids** ***n*** **(%)**	41 (75.9)	41 (75.9)	1	43 (79.6)	0.64
**Magnesium Sulphate,** ***n*** **(%)**	21 (38.8)	20 (37.0)	0.84	29 (53.7)	0.12
**Smoking,** ***n*** **(%)**	3 (5.5)	1 (1.8)	0.30	2 (3.7)	0.64
**Preeclampsia,** ***n*** **(%)**	22 (40.7)	2 (3.7)	<0.001	6 (11.1)	<0.001
**Twin pregnancy,** ***n*** **(%)**	19 (35.1)	16 (29.6)	0.53	18 (33.3)	0.83
**Male sex,** ***n*** **(%)**	26 (48.1)	26 (48.1)	1	26 (48.1)	1
**GA, mean (SD)**	31 (3.3)	31.1 (3.3)	0.80	29.6 (3.5)	0.03
**BW, grams, mean (SD)**	1327 (547)	1838 (606)	<0.001	1524 (572)	0.07
**Customised BW Centile, median (IQR)**	0.5 (2.5)	33.1 (39.3)	<0.001	31.7 (36.2)	<0.001
**HC at birth, cm, mean (SD)**	27.3 (3.5)	29.6 (3.1)	<0.001	28.1 (3.4)	0.29
**Length at birth, cm, mean (SD)**	38.6 (5.8)	42.7 (4.7)	<0.001	40.3 (5.1)	0.10
**Mode of delivery,** ***n*** **(%)**			0.79		0.55
Normal Vaginal	6 (11.1)	7 (12.9)		10 (18.5)	
Assisted Vaginal	1 (1.8)	2 (3.7)		1 (1.8)	
C/section	47 (87.0)	45 (83.3)		43 (79.6)	
**Apgar at 5** **min, median (IQR)**	9 (1)	9 (1)	1	9 (1)	1
Apgar at 5 min, ≤ **7,** ***n*** **(%)**	11 (20.3)	10 (18.5)	0.80	13 (24)	

We used Chi-squared test for categorical variables, *t*-test for parametric continues variables, Wilcoxon Rank-Sum test for non-parametric continuous variables.

*GA* gestational age, *BW* birthweight, *BMI* body mass index, *FGR* foetal growth restriction, *US* ultrasound, *HC* head circumference, *c/section* caesarean section.

In terms of neonatal outcomes (Table 2), preterm SGA infants were more likely to have hypothermia (44.4%) on admission to the nursery when compared with both gestational age matched controls (15.0%) and birthweight-matched controls (17.2%). Infants in the SGA group had significantly lower lowest BSLs at birth (mean=2.5 mmol/l, SD = 1.0) and in the first week of life (mean=3.8 mmol/l, SD = 1.1) compared with the birthweight-matched control group (mean=3 mmol/l, SD = 1.3 and 4.3 mmol/l, SD = 1.2 respectively). The proportion of infants who had hypoglycaemia were also more common in the SGA group, especially in the first week of life, compared with both control groups. SGA infants had higher lactate levels at birth (day 1), and between days 2 and 7, compared to both controls. No differences between groups were seen in the blood pH at birth or during the first week of life.

**Table 2:** Neonatal outcomes.

Neonatal Outcomes	Study Group (SGA)	Gestational matched Control Group (AGA)	Birthweight matched Control Group (AGA)
**Number of infants**	54	54	54
**Hypothermia on admission (temp. < 36 °C),** ***n*** **(%)**	24 (44.4)	8 (15.4) *	9 (17.3) *
**Lowest pH day 1, mean (SD)**	7.22 (0.09) n: 52	7.24 (0.08) n: 53	7.23 (0.08) n: 53
**Lowest pH day 2-7, mean (SD)**	7.28 (0.09) n: 51	7.29 (0.07) n: 52	7.27 (0.08) n: 52
**Lowest BSL day 1 (mmol/l) mean (SD)**	2.5 (1.0)	2.9 (1.2)	3.0 (1.3) *
**Hypoglycaemia (BSL < 2.6** **mmol/l) day 1** ***n*** **(%)**	27 (50)	21 (38.8)	21 (38.9)
**Lowest BSL day 2-7 (mmol/l) mean (SD)**	3.8 (1.1)	4.0 (0.9)	4.3 (1.2) *
**Hypoglycaemia (BSL < 2.6** **mmol/l) day 2–7,** ***n*** **(%)**	7 (13.7)	0 *	0 *
**Highest lactate day 1, mean (SD)**	5.2 (2.9) n: 53	4.2 (2.3) n: 52	4.1 (2.2) * n: 53
**Highest lactate day 2-7, mean (SD)**	2.9 (1.3) n:51	2.4 (1.0) * n:51	2.5 (1.0) n:51
**Respiratory support required n (%)**	39 (72.2)	35 (64.8)	44 (81.4)
**Mechanical Ventilation,** ***n*** **(%)** **days, median (IQR)**	15 (27.7) 7 (5)	14 (25.9) 4 (4)	24 (44.4) 5 (8.5)
**CPAP/HF/NIMV,** ***n*** **(%),** **days, median (IQR)**	37 (68.5) 25 (43)	35 (64.8) 17 (49)	44 (81.4) 38.5 (48.5)
**Low flow air/oxygen,** ***n*** **(%),** **days, mean (SD)**	17 (31.4) 10.5 (10.0)	14 (25.9) 11.7 (7.5)	23 (42.6) 14.5 (9.4)
**Respiratory support at day 28,** ***n*** **(%)**	18 (33.3)	19 (35.1)	29 (53.7) *
**BPD (oxygen at 36 weeks CA)** ***n*** **(%)**	15 (30.0)	11 (20.3)	22 (40.7)
**Home oxygen,** ***n*** **(%)**	3 (5.5)	2 (3.7)	6 (11.1)
**Inotropic support** ***n*** **(%)**	8 (14.8)	4 (7.4)	9 (16.6)
**Minimum HR Day 1**	128 (11.1)	125 (13.7)	129 (14.5)
**Maximum HR Day 1**	161 (12.5)	162 (14.1)	167 (14.3) *
**NGT feeds** ***n*** **(%)**	50 (92.6)	45 (83.3)	48 (88.8)
**NGT feeds (days) median (IQR)**	34 (38)	30 (41)	49 (41)
**Regain BW (days) mean (SD)**	8 (3.0)	11 (3.6) *	11 (3.6) *
**IVH** ***n*** **(%)**	5 (14.2)	6 (22.2)	15 (39.5) *
**PVL** ***n*** **(%)**	2 (5.7)	2 (7.4)	3 (7.9)
**NEC** ***n*** **(%)**	6 (11.1)	1 (1.8) *	3 (5.5)
**ROP** ***n*** **(%)**	4 (22.2)	6 (35.3)	11 (42.3)
**Death** ***n*** **(%)**	4 (7.4)	1 (1.8)	1 (1.8)
**Days in hospital, median (IQR)**	36.5 (41)	31 (50)	49 (53)
**GA at discharged, median (IQR)**	37 (1)	37 (1)	37 (2)

We used Chi-squared test for categorical variables, t-test for parametric continues variables, Wilcoxon Rank-Sum test for non-parametric continuous variables. *SGA* small for gestational age, *AGA* adequate for gestational age, *BW* birthweight, *GA* gestational age, *BSL* blood sugar level, *CPAP* continuous positive airway pressure, *HF* high flow, *NIMV* Non-Invasive Mechanical Ventilation, *BPD* bronchopulmonary dysplasia, *NGT* nasogastric tube, *IVH* intraventricular haemorrhage, *PVL* periventricular leukomalacia, *NEC* necrotising enterocolitis, *ROP* retinopathy of prematurity.

*p = ≤0.05 (highlighted in bold).

Most infants in all groups required respiratory support, and there was no significant difference in the steroid therapy received between groups. Although the differences in types of respiratory support were not statistically significant, there was a trend suggesting that mechanical ventilation was more frequently used in the birthweight-matched control group (with lower gestational age) compared to the SGA group (*p* = 0.07). Similarly, while not statistically significant, the incidence of bronchopulmonary dysplasia (BPD) was higher in the lower gestational age group (40.7%) than in the SGA group (30%) (*p* = 0.15). The requirement for inotropic support was similar between all groups. No differences were seen in maximum or minimum heart rates, except a higher maximum heart rate on day 1 in the birthweight-matched control compared to the SGA group.

SGA infants recovered to their birthweight faster (day 8 ± 3) than both control groups (day 11 ± 3.6). All groups had a reduction in z-score of weight, length and head circumference at discharge (Table 3). The changes in the z-scores of weights from birth to discharge were significantly smaller in the SGA group than in both control groups. There were also significantly smaller changes in z-scores of head circumference in the SGA group compared with gestational-matched control.

**Table 3:** Growth data.

Growth Data	Study Group (SGA) Mean (SD)	Gestational matched Control Group (AGA) Mean (SD)	Birthweight matched Control Group (AGA) Mean (SD)
BW (grams)	1327 (546)	1837 (605) *	1523 (571)
BW Percentile	15 (12)	61 (17)	57 (18)
DW (grams)	2431.6 (439)	2702.4 (409) *	2948 (935) *
DW Percentile	0.8 (0.8)	28 (17) *	30 (21) *
z-score of BW	-1.1 (0.62)	0.3 (0.50) *	0.2 (0.53) *
z-score of DW	-1.6 (0.71)	-0.6 (0.56) *	-0.6 (0.69) *
Difference in z-score of weight	-0.5 (0.47)	-0.9 (0.55) *	-0.8 (0.75) *
z-score of length at birth	-0.8 (1.1)	0.7 (0.7) *	0.5 (0.7) *
z-score of length at discharge	-1.5 (1.0)	-0.2 (1.0) *	-0.5 (1.0) *
Difference in z-score of length	-0.8 (1.0)	-0.9 (0.8)	-1.0 (0.7)
z-score of HC at birth	-0.8 (0.9)	0.6 (0.7) *	0.5 (0.6) *
z-score of HC at discharge	-1.1 (0.7)	-0.2 (0.7) *	-0.1 (0.7) *
Difference in z-score of HC	-0.4 (0.7)	-0.9 (0.7) *	-0.6 (0.9)

We used T-test for parametric continues variables. *SGA*, small for gestational age; *AGA*, adequate for gestational age; *BW* birthweight, *DW* discharged weight, *HC* head circumference. Difference in z-score of weight = z-score of DW – z-score of BW. Difference in z-score of length = z-score of length at discharge – z-score of length at birth. Difference in z-score of HC = z-score of HC at discharge – z-score of HC at birth. Percentiles and z- scores were calculated using the Fenton Chart.

*p = ≤0.05.

In terms of the neonatal outcomes (Table 2), NEC was significantly increased in the SGA group (11.0% vs. 1.8% (gestation-matched control)). SGA infants had a six-fold higher risk of NEC in our study (Table 4). All six cases of SGA infants who experienced NEC occurred in infants born at less than 28 weeks gestation. It is important to mention that the feeding practices within the SGA and the birthweight-matched control group were similar. Our local feeding practice in preterm infants involves using the mother’s own milk or pasteurised donor breast milk, early feeding, and a standardised feeding protocol based on birthweight. IVH was significantly more prevalent in the birthweight-matched control (39.5% versus SGA 15%) who were born at significantly earlier gestational ages compared with SGA cases. There were no significant differences in PVL and ROP between groups.

**Table 4:** Odds of Neonatal Outcomes for SGA infants compared to controls.

	SGA n	GA matched Control n	OR [95% CI]	p	BW matched Control n	OR [95% CI]	p
Hypothermia	24/54	8/54	4.4 [1.61–12.74]	0.0011	9/54	3.82 [1.44–10.60]	0.0026
IVH	5/54	6/54	0.58 [0.12–2.65]	0.41	15/54	0.25 [0.06–0.89]	0.01
PVL	2/54	2/54	0.75 [0.05–11.17]	0.78	3/54	0.70 [0.05–6.61]	0.71
NEC	6/54	1/54	6.62 [0.75–310.31]	0.05	3/54	2.12 [0.42–13.77]	0.29
ROP	4/54	6/54	0.52 [0.08–2.92]	0.39	11/54	0.38 [0.07–1.76]	0.16
Death	4/54	1/54	4.24 [0.39–212.63]	0.16	1/54	4.24 [0.39–212.63]	0.16

We used Logistic regression to calculate *OR* odds ratio and *CI* confidence interval, *n* Number of infants in each outcome, *SGA* small for gestational age, *GA* gestational age, *BW* birthweight, *IVH* intraventricular haemorrhage, *PVL* periventricular leukomalacia, *NEC* necrotising enterocolitis, *ROP* retinopathy of prematurity.

Length of hospital stay and the postmenstrual age at discharge were similar between groups. Five infants (3.3%) died before hospital discharge. Four deaths were in the SGA group (7.4%) compared to one control participant (1.8%, *p* = 0.17). All infants who died were born at or before 28 weeks of gestation. The causes of death were sepsis, NEC and chronic lung disease.

## Discussion

Our findings reveal that SGA preterm infants are at higher risk of hypothermia, hypoglycaemia and NEC, compared to their gestation- and birthweight-matched, well-grown, preterm counterparts. In contrast, IVH is more closely associated with gestational age. The SGA cohort really did reflect a cohort of true FGR, as evidenced by the very high rates of Doppler abnormalities (78.4%) and birthweight percentile less than 3^rd^ (80%).

Despite all efforts to prevent neonatal hypothermia after birth such as higher delivery room temperature, the use of polyethylene occlusive suits/bags in infants less than 32 weeks, warmed humidified resuscitation gases and the use of transport incubators, it remains common, especially in the preterm population of SGA infants. This is due to a relatively large body-surface-area and decreased body and subcutaneous tissue fat, impaired thermoregulation and catecholamine depletion [15]. Neonatal hypothermia is important to recognise because it is associated with increased neonatal morbidities and mortality [16]. A large retrospective cohort of late preterm infants found that infant birthweight was the biggest risk factor for hypothermia, ahead of gestational age [17]. Our results challenge these findings, indicating that perhaps the most important predictor is the birthweight centile, rather than birthweight and gestational age, which are adjusted for each other when the birthweight centile is derived.

Hypoglycaemia was also more common in SGA infants during the first days after birth, in keeping with many previous studies. It is due to decreased glycogen and fat stores, inappropriate release of insulin, and impaired counter regulatory hormones [18]. In our study, SGA infants had more episodes of hypoglycaemia as well as lower BSL levels than controls. Our SGA infants also demonstrated higher serum lactate levels during the first week of life. This likely reflects placental dysfunction as the cause of FGR, with relatively less oxygenation and a greater reliance on anaerobic metabolism to maintain a normal pH prior to birth.

Respiratory outcomes in FGR remain a grey area in neonatology. Traditionally, SGA infants have been assumed to demonstrate accelerated maturation due to intrauterine stress and thus are likely to experience fewer short-term complications of prematurity and less respiratory morbidity [19]. In contrast, more recent literature suggests that these infants have higher rates of BPD, and one study suggests that impaired vasculogenesis may be a contributory factor to the higher incidence of BPD in this population [20]. As well, higher rates of respiratory distress syndrome, pulmonary hypertension and meconium aspiration syndrome have been linked to SGA infants [21]. Sassi et al. described nearly doubled rates of home oxygen, three times the duration of ventilation, and ten times longer need for supplemental oxygen in FGR/SGA < 32 weeks’ gestation preterm infants, compared to their normally grown peers [22]. In our study we did not find a significant difference in the duration of ventilation support or the type of ventilation between groups. We postulate that this could be related to lack of power as well as the rather sizable difference in birthweight between the SGA and birthweight-matched groups. Our study suggests that infants born prematurely are at similar risk of respiratory morbidity *regardless* of SGA status. Similarly, infants born small are at similar risk of respiratory morbidity whether small from prematurity, or from being SGA.

The data on cardiovascular function is also conflicting. Echocardiogram assessments during the early postnatal period suggest that very preterm infants with FGR have altered cardiac geometry and function, with globular and hypertrophied hearts [23]. Disturbance in the autonomic nervous system and decreased heart rate variability have also been described in SGA infants. These have been explained by a less mature autonomic activity with higher sympathetic and lower vagal tone [24]. The predominance of sympathetic activity is strongly related to hypertension and other risk factors for cardiovascular disease. Thus, these findings could explain the risk of cardiovascular diseases in this population [25]. Although we did not assess heart rate variability in our study, differences between groups in the maximum and the minimum heart rate and the use in inotropic support were not observed. This may reflect that there is increased sympathetic tone both earlier in gestation, as well as in the setting of FGR.

Conflicting evidence has accumulated regarding risk of IVH in the SGA population, with some studies showing no increased risk or even lower risk [19, 26–28] and another showing a clear association [29]. The heterogeneity in those studies related to the diagnosis of FGR could explain these discrepancies [30]. It is clinically informative that our study has now matched for both gestation and birthweight to clearly show that IVH is more related to lower gestational age at birth.

Our finding that SGA is the leading risk factor for NEC is strongly supported by the literature. NEC has been significantly associated with SGA infants in many studies [26, 29, 31], along with early feeding intolerance [32]. Chronic foetal hypoxia and subsequent cardiovascular redistribution of blood flow away from the gastrointestinal tract is a likely a contributing factor for NEC [33]. Accordingly, how and when to start feeding in this population at high risk for NEC is still a hot topic in neonatology. Delaying the introduction of enteral feeds does not decrease the incidence of NEC. Moreover, starting feeds early in preterm life is associated with a reduction in cholestatic jaundice and improved weight gain [34]. Kempley et al. suggested that FGR infants less than 29 weeks may require an increased duration of minimal enteral feeds and a slower rate of feed advancement to facilitate gut adaptation, especially those with abnormal antenatal Dopplers [35].

Optimum growth in preterm SGA infants is still not well understood, but higher growth velocity in the neonatal period is associated with better neurodevelopmental outcomes at 18–22 months corrected age [36]. A previous cohort study showed that preterm SGA infants (birthweight <10^th^ percentile on Fenton Charts) experienced a smaller reduction in their weight z-score when compared with non-SGA peers where both groups had the same feeding regimen [37]. In a large growth cohort study, SGA infants lost a lower average percentage of weight postnatally than AGA and large for gestational age infants. Subsequently, the SGA infants regained their birthweight 4 days earlier than the AGA infants, and were approximately three times more likely to regain their birthweight percentile by discharge, compared to the non-SGA infants [38]. These findings are like ours and suggest that preterm SGA infants have a different growth velocity to well grown infants. The impact of this on later outcomes will be important to discern.

Many studies have described higher risk of death in the SGA preterm population compared with well grown preterm infants [26–29, 31]; one study reported an increase of nearly three-fold in the odds of mortality [29] – similar to what we have seen in our cohort. The Trial of Randomised Umbilical and Foetal Flow in Europe (TRUFFLE) recruited 503 women with a diagnosis of early-onset FGR. Infants were subsequently born with a mean gestational age of 30^+5^ weeks and outcomes described were better than expected from previous reports. Neonatal mortality was 6% (similar to the 7% seen in our cohort) and 70% survived without severe neonatal morbidity [39]. That our mortality figures are in keeping with these previous studies speaks to the generalisability of our findings overall. Larger studies also demonstrate that within each week from 25 to 32 weeks gestation, mortality and neonatal morbidities decrease with advancing gestation at birth [27]. These data inform perinatal decision-making for high-risk fetuses. However, a more nuanced assessment in the setting of FGR is possible. Like others [40], our study may aid improved and more individualised antenatal counselling and shared decision-making with parents regarding short and long-term neonatal outcomes, depending on whether the premature infant is or is not SGA.

The strength of our study includes a unique design matching SGA infant to gestational age and birthweight, as well as relatively good size sample for a single centre. However, this study had some limitations. Although not statistically significant (*p* = 0.07), the mean birthweight in the SGA group was 200 grams lower than that of the birthweight-matched control, which may minimise the difference in the outcome results.

## Conclusion

When we compare to both gestation-matched and birthweight-matched controls, preterm SGA infants have increased complications of hypothermia, hypoglycaemia and NEC. In contrast, intraventricular haemorrhage – a major cause of neurodevelopment disability - is more related to gestational age at birth and is not increased among the SGA. Early iatrogenic delivery which is performed to prevent stillbirth must be weighed against potential risks associated with prematurity in growth-restricted fetuses.

## Data Availability

The data supporting this study’s finding are available from the corresponding author, upon reasonable request.
